# A new perspective of the chemistry and kinetics of inactivation of COVID -19 coronavirus aerosols

**DOI:** 10.2217/fvl-2020-0326

**Published:** 2021-01-07

**Authors:** Michael L Hitchman

**Affiliations:** ^1^Department of Pure & Applied Chemistry, University of Strathclyde, Glasgow G1 1XL, UK

**Keywords:** aerosols, COVID-19, enveloped viruses, inactivation kinetics, inactivation reactor system, lipid bilayers, molecular collisions, parabolic optics, UV germicidal irradiation

## Abstract

In this paper we present a new approach to the mechanism of inactivation of enveloped virus aerosols. The analysis is in terms of oxidation of the lipid bilayer of the viral envelope through a free radical chain reaction. The rate kinetics of the process for various enveloped viruses have been compared and the indications are that the inactivations are closely related. Promoting virus inactivation with UV light is briefly reviewed and discussed as an extension of the chain reaction mechanism, which with physicochemical analyses give insights into the process and of reaction complexities. An outline of a practical method of achieving a 3-log10 level of deactivation in 1 min is described with purified air being returned to healthcare environments.

The current COVID-19 pandemic has led to hundreds of papers and articles about the causative agent, pathogenesis and immune responses, epidemiology, diagnosis, treatment and management of the contagion, and control and prevention strategies. There have been rapid advances on various aspects of the disease. One particular area of study has been of the transmission of the virus and of its survival in medical environments [1,2]. However, there is still much that is poorly understood and there is a need not just for clinically based comprehension of virus transferal, but also for a more general approach using physicochemical measures to give new insights into viral epidemiology. In this paper, we use different perspectives to try and understand the chemistry of COVID-19 aerosols and of viral inactivation.

A recent report by van Doremalen *et al.* has analyzed the aerosol and surface stability of SARS-CoV-2 and compared it with SARS-CoV-1, and their decay rates have been estimated [3]. The authors found that, under the experimental conditions tested, the stabilities of the two viruses were similar, and they concluded that the results indicated that transmission of SARS-CoV-2 within an aerosol droplet is plausible since the virus can remain viable and infectious for significant periods. Here we consider, for the first time, chemistry for viral inactivation, and analyze associated physicochemical processes which give insights into rapid viability reduction by orders of magnitude.

## Virus inactivation

Plots of titers as log (TCID_50_)/liter of air against time showed exponential decays for both SARS-CoV-2 and SARS-CoV-1 [3]. That clearly indicates that the inactivation process involves a first order, or pseudo-first-order reaction. Let us consider what that might be.

As is well known, in some viruses the capsid of the virion with its enclosed nucleic acid is surrounded by a lipoprotein envelope, as in coronaviruses. The bilayer envelope of those viruses is derived from portions of host cell membranes and includes anionic lipids [4]. The role of lipids in virus replication is inevitably intricate and complex [4,5]. The envelope has many functions in viral infection, including virus attachment to cells, entry into cells, the release of the capsid contents into the cells and packaging of newly formed viral particles [6]. To try and elucidate those roles, studies have been made of the composition of viral envelopes, for example, through the development of lipodomics, a mass spectrometry-based systematic analysis of cellular lipids in general [7]. And although it has been recognized that the lipid structure is also responsible for the stability characteristics of the virus particle, such as resistance to chemical or physical inactivation [6], the structural studies have focused on their interaction with other molecules, their cellular functions and analysis of glycerophospholipids as a tool for the assessment of species specific biomarkers of viral pathogenicity [8].

**Figure 1.**
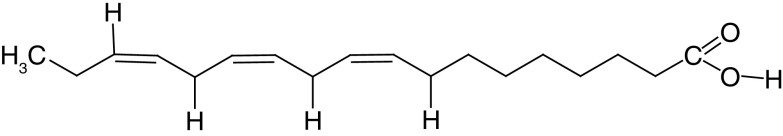
Typical structure of a fatty-acid lipid.

In order to rationally enhance virus inactivation, it would be desirable to understand lipid chemistry and reactivity. Here we take a very simple chemical approach in order to provide an appreciation of viral aerosol deactivation. And we start by considering a typical fatty-acid lipid structure in isolation (Figure 1).

Enveloped viruses can persist outside host environments typically for days, while nonenveloped viruses can survive for weeks [9,10]. Therefore, using the principle of Occam’s razor, it would not be unreasonable to suggest that observation could be related to the relative ease with which the lipid structure is disrupted. The most common mechanism of the degradation of lipids is environmental oxidation through the effects of light, heat, humidity and, of course, aerial oxygen, all of which can accelerate the breakdown of the lipid molecular chain, and that oxidation occurs wherever unsaturated fatty-acid lipids are found [11,12].

The process by which lipid oxidation occurs has, in general, long been recognized as a free radical chain reaction with the classic stages of initiation, propagation and termination leading to a series of complex chemical changes. Here, we will illustrate the process in a greatly simplified scheme in order to try and discover what could be involved in viral deactivation.

In the presence of an environmental initiator, such as light [13], the viral envelope lipid loses a hydrogen atom and produces free radicals. In the case of the inactivation study by van Doremalen *et al.* [3], the aerosols were kept in a Goldberg drum with no light access, but during their generation using a nebulizer it is probable that viable viruses would have been exposed to daylight or artificial light. Both light sources have UV components. Daylight with the sun at zenith can consist of up to 3% UV light of which 95% is UVA [14], and that light from ca 315–400 nm has sufficient energy of 378–299 kJ/mol to initiate free radical formation from a lipid, as indicated below. Artificial light, particularly fluorescent lighting has lower levels of UVA, but it is still sufficient to interact with organic structures [15].

We represent a single viral lipid as VL_1_H, and initiation, the formation of an ab initio viral lipid free radical (VL_1_^•^), can be represented by:

(Eq. R1) $${\text{VL}}_{\text{1}}\text{H}\overset{\text{Removal of H}}{\to }{\text{VL}}_{\text{1}}^{•}$$

Low C–H bond energies (322 kJ/mol) in unsaturated fatty-acid lipids are found for the allylic hydrogens next to double bonds (cf. Figure 1; H_3_C–CH_2_–CH=CH–) and these are preferred sites for H removal and formation of a free radical [11]. The C–H bond energies for the –CH_2_– groups between two double bonds in lipids (called doubly allylic, –HC=CH–CH_2_–CH=CH–) are more activated and C–H bond energies are even lower (272 kJ/mol) [13]; it can be noted that lipid dissociation will be aided by the associated increase in entropy. For lipids without double bonds, the formation of a free radical is more difficult for the bond energy of a non-allylic H atom is much higher (418 kJ/mol) [13].

The viral lipid radical reacts with aerial oxygen to form a peroxyl radical, VL_1_OO^•^,

(Eq. R2) $${\text{VL}}_{\text{1}}^{•}+{\text{O}}_{\text{2}}\to {\text{VL}}_{\text{1}}{\text{OO}}^{•}$$

which acts as the chain carrier for propagating the reaction by attacking an adjacent viral lipid, VL_2_H, to form another viral lipid radical (VL_2_^•^), and hydroperoxide (VL_1_OOH),

(Eq. R3) $${\text{VL}}_{\text{1}}{\text{OO}}^{•}+{\text{VL}}_{\text{2}}\text{H}\to {\text{VL}}_{\text{1}}\text{OOH}+{\text{VL}}_{\text{2}}^{•}$$

followed by a series of reactions:

(Eq. R4) $${\text{VL}}_{2}^{•}+{\text{O}}_{2}\to {\text{VL}}_{\text{2}}{\text{OO}}^{•}$$

(Eq. R5) $${\text{VL}}_{\text{2}}{\text{OO}}^{•}+{\text{VL}}_{3}\text{H}\to {\text{VL}}_{\text{2}}\text{OOH}+{\text{VL}}_{3}^{•}$$

and so on. The free radical chain reaction is thus established.

From a single initiation this reaction can be repeated many times during propagation until termination when no hydrogen source is available or radical scavenging becomes excessive. Therefore, viral lipid oxidation is self-propagating. As indicated above, the process is rather more complex, and one example of that complexity will be discussed further below when we consider initiation by higher levels of UV light.

Since the proteins on the surface of the envelope serve to identify and bind to receptor sites on the host’s membrane when the viral envelope fuses with it, allowing the capsid and viral genome to enter and infect the host, it would not be surprising that extensive oxidative rupturing of the lipid structure would disturb infection transmission.

It is worth noting in that context that free radicals from lipid oxidation can also undergo co-oxidation with proteins since hydrogens are readily available for abstraction on side chain amino and thiol groups of amino acid constituents [16]; for example:

(Eq. R6) $${\text{VLOO}}^{•}+-\text{NH2}\to \text{VLOOH}+-{\text{NH}}^{•}$$

(Eq. R7) $${\text{VLOO}}^{•}+-\text{SH}\to \text{VLOOH}+-{\text{S}}^{•}$$

Such reactions with spike proteins will also clearly add to virus inactivation.

## Kinetics of virus inactivation

Although there is a series of steps (Eq.R1) to (Eq.R5), the overall oxidation can be simply written as:

(Eq. R8) $$\text{VLH}+{\text{O}}_{2}\to \text{VLOOH}$$

to form a hydroperoxide, VLOOH.

The hydroperoxides are the predominant oxidation products and are relatively stable, so they can build up with time [17,18]; cf. reactions (R3–R5). Hence their formation can be treated as coming from a bimolecular oxidation and we can use reaction (R8) as the overarching process.

A viral hydroperoxide will have a very different structure from the initial viral lipid, VLH, and would be expected to induce a different viral activity and to impede infection transmission; in other words, viral deactivation will occur. The rate at which that happens can then be described by:

(Eq. 1) $$d\left[VLH\right]/dt=-k_{2}\left[VLH\right]\times \left[O_{2}\right]$$

Square brackets indicate concentrations, and this differential equation gives the rate of disappearance of the active viral lipid VLH, often with units of mol/l/s; other measures of concentration and time can obviously be used. The constant, k_2_, is the constant of proportionality of the rate with the reactant concentrations. Typically, it will have the units of l/mol/s as is usual for a process involving two reactants; in other words, a second-order reaction. We now compare virus and oxygen concentrations.

The initial infectious virus titers measured by van Doremalen *et al.* [3] were 10^3.5^ TCID_50_ per liter of air for SARS-CoV-2 and 10^4.3^ TCID_50_/l for SARS-CoV-1, or an average of approximately 10^4^ TCID_50_/l; the mean number of infectious units per volume (PFU/l) can be obtained by applying the Poisson distribution and taking 0.7 × TCID_50_ titer/l.

The virions are not just bare units though, but, as mentioned above, are contained in aerosol droplets. The aerosols were <5 μm [3] and a typical volume will be approximately 6 × 10^-14^ l. Aerial oxygen dissolved in an aqueous aerosol will be approximately 10 mg/l [19]. So in an aerosol droplet there will be approximately 2 × 10^-17^ moles of oxygen or approximately 10^7^ molecules. Let us assume for the moment that each aerosol droplet contains only one virion. In a lipid bilayer there are about 5 × 10^6^ lipid molecules in a surface of 1 μm × 1 μm [20], or a surface concentration of approximately 5 × 10^18^ molecules/m^2^.

A virion has a diameter of about 100 nm [21] and the lipid envelope surface area will be approximately 3 × 10^-14^ m^2^. Thus, there will be approximately 10^5^ lipid molecules on the virion envelope and there will be an excess of approximately 100 molecules of oxygen in the aerosol droplet to bring about virion deactivation. Overall, there are approximately 10^4^ infectious units per liter of air so there will be approximately 10^9^ lipid molecules for oxidation, again with a 100-fold excess of dissolved oxygen. That may not seem a very large excess, but we also have to take account of the replenishment of the oxygen from the surrounding air. Assuming ideal gas behavior, one liter of air at standard temperature and pressure (STP) will contain approximately 10^21.7^ oxygen molecules so there will be a very large excess available. And the time for aerial oxygen to dissolve and reach the virion surface where it is consumed will be a matter of milliseconds; the diffusion coefficient of oxygen in water is approximately 10^-5^ cm^2^/s and the time taken for diffusion across an aerosol length of 2.5 μm is (2.5 × 10^-4^)^2^/10^-5^ ≈ 6 ms [19] which, as indicated below, is miniscule compared with the oxidation rate. Even though there will be more than one site in a lipid molecule where oxidation can occur (cf. Figure 1), and there may be a greater number of virions in each aerosol droplet, the dissolved oxygen will never be depleted.

It should also be noted that for real-life situations aerosol droplets generally are much larger than 5 μm. For sneezing, droplet sizes can be as large as 360 μm, while for coughing and breathing they can be 60–100 μm in diameter, and practically no droplets have diameters <3 μm [22]. Notwithstanding the wide range of droplet sizes, oxygen will always be the dominant reactant.

Therefore, in reaction (R8) the [O_2_] will effectively stay constant and Eq. (1) can be written as:

(Eq. 2) d[VLH]/dt=-k‘[VLH]

where the rate constant, k’, contains the oxygen concentration and has the units of reciprocal time; for example, s^-1^; it is a pseudo-first order rate constant. Integration of Eq. (2) shows the exponential decay of the virus concentration with time mentioned above:

(Eq. 3) $$\left[VLH\right]={\left[VLH\right]}_{0}exp\left(-k‘t\right)$$

To halve the initial concentration, [VLH]_0_, the time, t_1/2_, for SARS-CoV-2 is 1.1 h, or 3960 s, and for SARS-CoV-1 it is 1.2 h, or 4320 s [3]; these reaction times are clearly very much longer than times for replenishment of the oxygen dissolved in the aerosol droplet.

The relationship between a pseudo-first order rate constant and a half-life is given by

(Eq. 4) $$k’=ln\;2/\left(t_{1/2}\right)$$

so the two rate constants are 1.75 × 10^-4^/s and 1.60 × 10^-4^/s, respectively. The kinetics of the inactivation of the two viruses is obviously very similar.

As mentioned above, published comparisons of enveloped viruses have been mainly related to medical considerations such as transmissibility, hospitalization, mortality rates, pathogenesis, epidemiology and other clinical features. None seem to have considered details of the chemical structure of the viral envelopes. However, there is circumstantial evidence that suggests there are similarities for a range of viral envelopes.

For example, values of t_1/2_ of about 840, 1140 and 4800 s have been found for H1N1, H5N1 and H3N2 virus aerosols, respectively [23], and 840–1140 s for African Swine Fever (ASF) virus aerosol [24]. In a study of the aerosol stability of Zaire Ebola virus, Fischer *et al.* [25] compared the viability over time of the two strains, EBOV Mayinga 1976 and EBOV Makona 2013. From their results the time required for a reduction of 1-log10 TCID_50_ was calculated to be 37140 s, which corresponds to a value of t_1/2_ of 11187 s. So values of t_1/2_ for a number of coronaviruses can be seen to lie in the range of 840–11187 s with SARS-CoV-2 and SARS-CoV-1 lying in midrange. Kramer *et al.* provide further evidence in comparing duration of persistence of >20 enveloped and nonenveloped viruses [9].

The relatively narrow range of half-lives suggests that, in general, enveloped structures are not too dissimilar and, hence, inactivations are comparable. The variations that do arise are not too surprising, though, when one takes into account the fact that viral lipid envelopes in different molecular environments will certainly have some variations in their structures, and, importantly, they will also experience different electron forces and distributions from the nucleocapsid that will affect their chemistry and reactivity; in that context it can be noted that there is only a small similarity between the complete genome sequences of COVID-19 and of Ebola [26].

Interestingly, a study of isolated phospholipid monolayers [27] without any viral environment and with various degrees of unsaturation has shown similar kinetic behavior to that for SARS. The degradation with time of different phospholipid molecules exposed to laboratory air was monitored. Remembering the discussion above about the effect of the proximity of double bonds in the lipid structure to the sites for hydrogen atom removal, it is interesting to see that a lipid with one double bond had a longer degradation t_1/2_ of 6120 s than the t_1/2_ of 5040 s for a lipid with two double bonds.

Some caution is needed, though, in making comparisons of t_1/2_ values. For example, the phospholipids were, as mentioned, exposed to laboratory air and hence to ambient lighting conditions, and, as we have already indicated and as we shall see further below, light can enhance the reaction kinetics. Then, as will also be discussed below, rates of lipid oxidation can vary depending on their physical state. For the phospholipid studies, monolayers were spread on a pure water surface of a Langmuir trough. Surface oxidations are generally slower than for aerosol samples so that may be why the rates of oxidation of simple molecules appear to be less rapid than for more complicated virus lipids. Notwithstanding experimental differences, there are similarities between the kinetic characteristics with comparable generic behavior of lipid molecules, irrespective of their environments. The usefulness of considering the chemistry of inactivation can be seen.

Before moving on to considering ways of enhancing virus inactivation we can note two additional features of the phospholipid study. First, a lipid with no double bonds showed no degradation which is consistent with the difficulty, discussed earlier, of initiating the chain reaction with no allylic hydrogens. Then, for the two-double-bonded lipid protected by a blanket of nitrogen there was no degradation, so confirming the role of aerial oxygen in the reaction process of reactions (R2) & (R4).

## Promoting virus inactivation

In order to have an indoor viral aerosol environment that is safe to use, the level of inactivation required clearly has to be defined. So, for example, if we choose that to be 99.9% requiring a 1000-fold reduction of the initial virus level then, by considering Eq. (3) with Eq. (4), it will need (ln1000/ln2) ≈ 10 half-lives. For SARS-CoV-2, assuming that the oxidation from initiation continues to follow first-order kinetics, that is, 11 h, which is not a realistic time to have to wait; a level of reduction to only 1% will still need approximately 8 h.

A number of studies has been published on inactivation of a wide range of viruses, in particular for enveloped viruses, which include treatment with steam, vaporized hydrogen peroxide, ozone and chlorine. However, to introduce any of those chemicals into a virus infected atmosphere without having an isolated environment, would give chemical contamination of the atmosphere and safety concerns. Another possibility is to use UV light.

A review of UV germicidal irradiation (UVGI) doses for coronavirus inactivation [28] comments that since coronaviruses have similar general structures, as we have indicated above, common UV disinfection procedures could inactivate the SARS-CoV-2 virus, as well as future possible mutations. However, that conclusion is based on UV damage to the ssRNA components which undoubtedly will occur, but the lipid bilayer presents a very much larger photon absorption cross-section that, as we shall see, can lead to sequential oxidation of hundreds of molecules in an exploding chain reaction.

The majority of the studies made has been with UVC light at 254 nm which presents its own hazards to skin and eyes, and it has been suggested that far-UVC light (207–222 nm) be used which efficiently kills pathogens with much less harm to exposed human cells or tissues [29,30]. That work has explored UV efficacy against human coronaviruses from subgroups alpha (HCoV-229E) and beta (HCoV-OC43). It was found that low doses of, respectively, 1.7 and 1.2 mJ/cm^2^ inactivated 99.9% of aerosolized alpha coronavirus, 229E, and beta coronavirus, OC43. The authors say that based on the results for the beta HCoV-OC43 coronavirus, continuous far-UVC exposure in public locations at the currently recommended exposure limit (3 mJ/cm^2^/h) would result in 99% viral inactivation in approximately 960 s and 99.9% inactivation in approximately 1500 s. It is suggested that it is realistic to expect that far-UVC light would show comparable inactivation efficiency against other human coronaviruses, including SARS-CoV-2; that is in accord with our discussion on viral lipid oxidation. The times for achieving that would still be too long though for a safe occupied environment. We consider here physicochemical aspects of dealing with that. To do that we need to first return to the chain reaction mechanism discussed above.

We noted earlier that peroxyl radicals (VL_1_OO^•^) act as chain carriers in oxidation, but we did not point out that the abstraction of reaction (R3):

(Eq. R3) $${\text{VL}}_{\text{1}}{\text{OO}}^{•}+{\text{VL}}_{\text{2}}\text{H}\to {\text{VL}}_{\text{1}}\text{OOH}+{\text{VL}}_{\text{2}}^{•}$$

is rather slow [12]. So, the chain can continue with just one abstraction at a time after initiation and the process can go on indefinitely at a slow rate. As a result, as we have noted, the hydroperoxides (VL_1_OOH) can build up with time. What is needed to accelerate it is for the hydroperoxides to be decomposed to alkoxyl radicals (VL_1_O^•^) and hydroxyl radicals (HO^•^) – reaction (R9):

(Eq. R9) $${\text{VL}}_{1}\text{OOH}\to {\text{VL}}_{1}{\text{O}}^{•}{+}^{•}\text{OH}$$

That is what UV light can do.

It can be noted that the cleavage of the O–O peroxide bond with an energy of 157 kJ/mol [12] will be even more favorable than that of any C–H bonds. Also, both the newly formed radicals react much more rapidly and more generally than VL_1_OO^•^. And since the homolytic scission by UV light creates two highly active radicals, two viral lipid oxidations will occur for each hydroperoxide decomposition. All of that means that one would expect there to be a significant increase in the rate of the overall chain propagation and lipid oxidation as the process progresses. In fact, reaction (R9) is an example of chain branching with secondary chains dramatically amplifying and extending the process beyond the initial radical chain; a single initiating event can lead to sequential oxidation of hundreds of molecules in the primary chain and in the secondary branching chains. The suggestions for the chemistry of enveloped virus inactivation give insights into how UV light can be used. However, there are results that suggest it is not straightforward.

**Figure 2.**
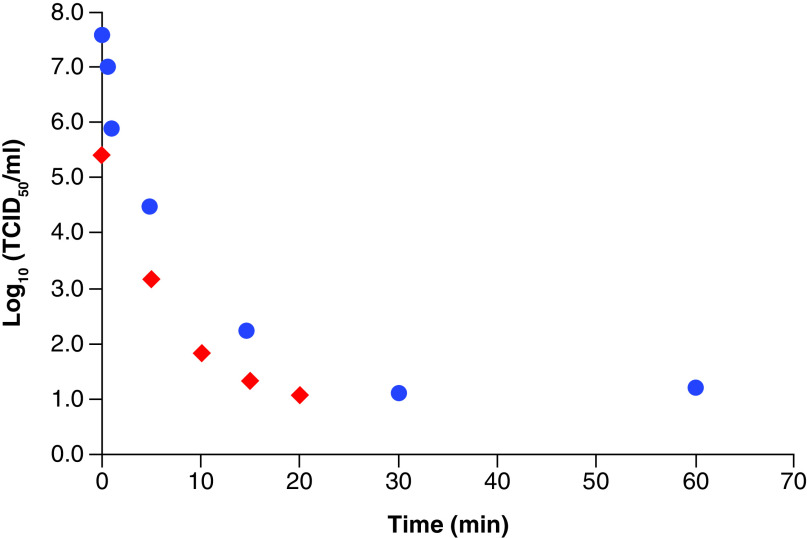
Log survival values for H1N1 virus (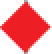) and SARS-CoV-1 (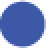) with time for inactivation by UV irradiation (254 nm). Data taken from Szeto *et al.* [31] and Kariwa *et al.* [32].

Figure 2 plots log reduction of virus titers (TCID_50_) for data taken from results for UV radiation of influenza virus H1N1 [31] and for SARS-CoV-1 [32]. In both cases the plots are initially approximately linear for a reduction of approximately 3-log10, and one can calculate the values of t_1/2_ for that region of the plots using Eqs. (3) and (4) +-03

For H1N1, t_1/2_ is approximately 42 s which is rather less than the ‘unassisted’ value of 840 s [23]. It should be noted, though, that the ‘unassisted’ result was measured with an aerosol sample and the UV-treated results were obtained with samples on nitrocellulose filter papers. As mentioned earlier, aerosol studies yield higher rate constants than for surface samples and this is because viruses tumbling in the air will receive all round UV exposure while those on surfaces receive exposure in one plane only, and virus samples absorbed into filter papers would be even less accessible to UV light. Therefore, a t_1/2_ for UV-irradiated H1N1 as an aerosol would be expected to be <42 s.

For SARS-CoV-1, t_1/2_ is approximately 30 s which is a significant decrease from the ‘unassisted’ value of 1.2 h, or 4320 s, reported above [3]. UV-irradiated SARS-CoV-1 consisted of 2 ml samples of stock virus in Petri dishes [32], which would give rather more light accessibility that would not be so different from the aerosols, so a significantly greater degree of inactivation can occur.

Even though there are some environmental and measurement factors that have to be taken into account, it can be seen that, in general, as is well known and as expected from considerations of chain propagation, UV light can be a powerful promoter of virus inactivation, at least initially. But then the temporal plots have decreasing slopes showing a slowing down of the inactivation processes.

One possible explanation for that rate falloff is that when radical quenching processes exceed the rate of new chain production, non-radical products form. An example of that is the co-oxidation mentioned earlier of non-lipid molecules such as the spike proteins; this can be a significant mechanism for radical scavenging and reducing lipid oxidation. And as oxidation progresses and the number of radicals dramatically increases, radical recombination can become more important and faster than the initiation of new chains so that oxidation slows as stable secondary products form. The chemistry changes and, hence, so does the kinetics.

However, there is a significant difference for aerosol viral lipid oxidation from that of more usual situations of lipid chain reactions in that, as we have seen and as indicated in Figure 2, virus concentrations are very low. And radical recombinations require not only high radical concentrations, but they also need a high viscosity medium. So when there is a high dilution of viral lipids and an aqueous aerosol environment, radical recombinations could be expected to be less important.

There is another way of considering the falloff in inactivation. We consider the number of collisions in a random way between molecular species [33]. A collision between a viral lipid and a photon will not necessarily lead, though, to acceleration of viral inactivation. The number of hydroperoxides decomposed in relation to the number of photons absorbed is known as the quantum yield, ϕ, and it is equal to the ratio of the measure of the probability that a photon will deactivate a virus as defined by the inactivation cross-section, σ, to the probability that a photon will be absorbed by a virus as defined by the absorption cross-section, A [34].

(Eq. 5) ϕ=σ/A

The quantum yield is an absolute measure of viral sensitivity to light. The relative sensitivity of different viruses varies [34], and, together with the factors mentioned above, that will be another reason for different inactivation half-lives.

Now, if we choose a particular virus it will have over a period of time, t, a number of inactivating collisions, nv, with photons. This can be expressed as:

(Eq. 6) nv=t/τ

where the proportionality constant, τ, is the average time between the inactivating collisions. If we initially have nv_0_ viruses in a given volume then after time t some will have experienced inactivating collisions, and we denote the number that have not had inactivating collisions until time t as nv_t_. The number nv_t+dt_ that then does not have an inactivating collision until time (t+dt), will be less than nv_t_ by the number of successful collisions in dt. The number of those collisions in dt can, using Eq. (6), be written as:

(Eq. 7) $$dnv=nv_{t}\;dt/\tau$$

Hence

(Eq. 8) $$nv_{t+dt}=nv_{t}-nv_{t}\;dt/\tau$$

The term nv_t+dt_ can be written as nv_t_ + (dnv_t_/dt) dt and substituting this into Eq. (8) gives

(Eq. 9) $$dnv_{t}/dt=-nv_{t}/\tau$$

Integrating Eq. (9) yields

(Eq. 10) $$nv_{t}=nv_{0}exp\left(-t/\tau \right)$$

Eq. (10) is of the same form as Eq. (3) with 1/τ being a frequency equivalent to the rate constant, k’, in Eq. (3). But whereas k' has a constant value in Eq. (3), 1/τ will be changing with time since τ is the average time between inactivating collisions which will get longer as time progresses because there are fewer viruses left that have not been inactivated. So not only is the number of viable viruses decreasing, but the number of collisions with photons to inactivate them is also decreasing.

This effect can be seen mathematically if we take the derivative with respect to time of the Ln of Eq. (10).

Then we have:

(Eq. 11) $$d\left(ln\left\{nv_{t}/nv_{0}\right\}\right)/dt=1/\tau$$

which shows that as τ increases, the slopes of the plots will tend to zero, as is observed in Figure 2 for the plots approaching the limit of detection.

Notwithstanding the falloff in the rate of viral lipid oxidation, the use of UV light can maintain an enhanced rate of inactivation for at least a reduction of 3-log10. Again, it is apparent that physicochemical considerations can lead to some understanding of viral inactivation. The question then is ‘How can that be implemented?’

## Use of UV light for inactivation

Germicidal UV lamps have been used for many years [35] with the main emission line for the UV radiation at 254 nm, which has 471 kJ/mol of energy. That will be sufficient to break the bonds we have been discussing Table 1.

**Table 1.** Lipid bond energies.

Bond		Energy (kJ/mol)
Allylic H	H_3_C–CH_2–_CH=CH–	322
Doubly allylic H	HC=CH–CH_2_–CH=CH–	272
Peroxide	O–O	184

Reports have been published for controlling hospital air quality and for analyses of UVGI for hospital applications [36,37] and detailed modeling of the UV dose has been carried out defining the complete 3D intensity field in any experimental apparatus involving airflow [38]. Here we present a simple overview of a reactor system which could be used to supplement other engineering control methods [37].

The first task is to estimate the light intensity for the UVGI application and this can be done with the aid of the relationship [39]:

(Eq. 12) $$nv_{t}/nv_{0}=exp\left(-k“I\;t\right)$$

where nv_t_ and nv_0_ are the number of surviving viruses at any time t and the initial number of viruses, respectively, as discussed in the previous section, k'' is equivalent to the rate constant in Eq. (3) but with units of cm^2^/μJ, and I is the UV radiation intensity in μJ/cm^2^ s. The rate constant k'' has to have units of cm^2^/μJ to ensure the argument of the exponential is dimensionless and it arises because k'' is a rate constant corresponding to a light intensity of 1 μJ/cm^2^ s; it is known as the standard rate constant and is independent of intensity.

For the Kariwa results [32] with the half-life given above of t_1/2_ approximately 30 s, the apparent rate constant from Eq. (4) is 0.023/s which is equal to k''I in Eq. (12). The light intensity is 134 μW/cm^2^ or 134 μJ/cm^2^ s [32] so k'' will be 0.023/134 = 1.7 × 10^-4^ cm^2^/μJ, and for a light intensity of 1 μJ/cm^2^ s that gives k' = 1.7 × 10^-4^/s which compares well with the value of the rate constant of 1.6 × 10^-4^/s obtained for SARS-CoV-1 from Eq. (4) and indicates that the ambient light that was mentioned for initiation of radical formation from the viral lipid had an energy of approximately 1 μW/cm^2^.

As just noted, k'' is independent of intensity, but with the additional energy input from the UV light the fractional level of viral inactivation will vary according to the magnitude of that input as described by

(Eq. 13) F=exp(-k”I t)

where the ratio nv_t_/nv_0_ in Eq. (12) is written as the survival fraction, F.

From Eq. (13), we have

(Eq. 14) 2.303  log F=k“I t

and putting in the numerical values with 3-log10 inactivation in 1 min and solving for I for SARS-CoV-2 gives

(Eq. 15) $$I=2.303\times 3/1.75\times 10^{-4}\times 60\approx 660\mu J/cm^{2}\;\;s=660\mu W/cm^{2}$$

Determining the air flow rates for healthcare environments is not straightforward, but a natural ventilation requirement rate of 216 m^3^/h/patient, or 60 l/s/patient, is recommended by the WHO [40]; other studies give support for that [41]. If a patient space such as a hospital bed in a ward or an intensive care unit (ICU) area has an air extraction unit above or in an enclosed adjacent space, contaminated air could be extracted and treated with UVC light. On that basis 3600 l, or 3.6 m^3^, would need to be extracted and to pass through the UV-irradiated region in a duct in the ceiling void or enclosed space every 60 s.

Welch *et al.* [29] have described a horizontal rectangular irradiation chamber through which a viral aerosol flows and passes over UV lights during part of their passage through the chamber. The ceiling duct would be a similar arrangement, but there would be multiple UV lamps at regular intervals along the whole length of the duct to give uniform irradiation of the air passing through it. Also, the duct, while having an inlet and outlet for the air flow, would be designed to ensure that no UV light could escape into any occupied environment; from that point of view it would not matter which type of UVC lamp was used.

The dimensions of the irradiated section of the duct could be adjusted to provide a volume of 3.6 m^3^, and the simplest arrangement would be to have a duct 3.6 m long and a cross sectional area of 1 m^2^. By introducing baffles along the length of the duct the path travelled by the airflow could be significantly enhanced and the cross-sectional area could be proportionately reduced to maintain the volumetric flow rate. Also, by having a serpentine sequence of separate duct sections the system would be less cumbersome. We now need to consider the radiation characteristics.

**Figure 3.**
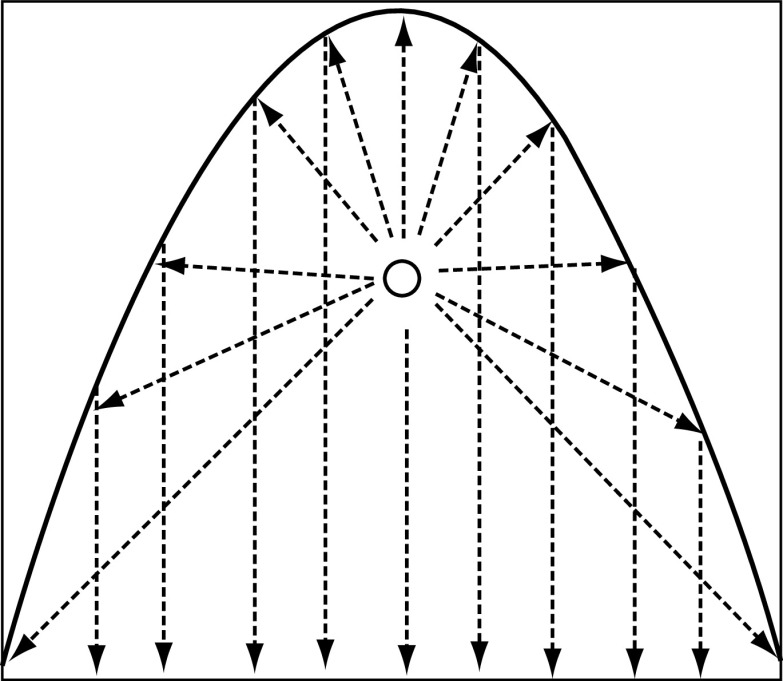
Schematic representation of a parabolic mirror in a duct for virus inactivation. The lamp denoted by O extends into the plane of the page.

Modeling of the UV intensity field due to enclosure reflectivity has generally been made for a rectangular duct with each of the four walls reflecting a fraction of the incident intensity it experiences at the surface [38]. An alternative geometry that would give more controlled and more intense irradiation would be for the duct to be in the form of a parabola. A line source of light, as is typically used for UV lamps, placed at the focus of a parabolic mirror will generate a cylindrical wave that will be reflected into a plane wave propagating as a collimated beam parallel to the vertical axis of the lamp (Figure 3).

Aluminum has a good reflectance (∼92%) in the UV spectral region [42]. It would not be used, though, in the usual form of a glass mirror since only special glasses have a high transmittance in the UV, and also fabricating a large parabolic glass mirror would be difficult and uneconomic. Aluminum evaporation is an industrially mature process and production of a mirror on a lightweight material that could be easily formed into a parabola would be straightforward. To compensate for the loss of light intensity by the reflection and to maintain the intensity required as calculated above we need to increase it to 660/0.92 ≈ 720 μW/cm^2^.

The dimensions of a symmetrical parabolic mirror can be related by the equation 4fh = r^2^, where f is the focal length, h is the height of the mirror measured along the axis of symmetry from the vertex to the plane of the rim (100 cm), and r is the radius of the mirror at the rim from the center (50 cm) [43]. With the mirror reaching from the base of the duct to the top and occupying the full width at the base then the focal length, f, will be 6.25 cm.

The fraction of light (θ) reflected by the dish from a light source in the focus, is given by [43].

(Eq. 16) $$\begin{array}{l} \theta =1-\frac{arctan\left(r/\left(h-f\right)\right)}{\pi }\\ \;\;\;\;=0.84 \end{array}$$

That requires a further increase in the light intensity to 720/0.84 ≈ 860 μW/cm^2^.

The cross-sectional area (A) under the parabola can be calculated by integrating the equation of a parabola

(Eq. 17) $$y=\alpha x^{2}$$

with the limits of the parabola width; α is a constant that can be determined from the fixed parabola dimensions. For r = 50 cm, A = 6666 cm^2^. We still have to maintain a volumetric flow rate of 3.6 m^3^/min, so the length of the parabolic duct will need to increase to approximately 5.4 m; the volume will still be treated in 1 min since while the volumetric velocity stays the same, the linear velocity increases as a result of the reduction in the cross-sectional area. One might also have a plane mirror at the base of the parabola to enhance the light intensity.

We take the total area for irradiating as the length of the parabolic duct times its maximum width which is 54 × 10^3^ cm^2^. Suitable lamps are conveniently available in 54 cm lengths so ten lamps would cover the whole length of the duct and each lamp would be required to provide 860 × 10^-6^ × 54 × 100 ∼ 5 W. It should be noted that UVC lights are about 35% efficient so the total wattage of each lamp needs to be 5/0.35 ≈ 14 W, which is also available. The total power for the system will be approximately 140 W, which is not excessive.

It should also be mentioned that germicidal lamps emit about 8% light at 185 nm producing ozone, which has been recognized for over 100 years as a viricide [44]. However, when inhaled, relatively low amounts of ozone can damage the lungs causing chest pain, coughing, shortness of breath and throat irritation [45]. Therefore, if UV-treated air is to be recirculated or to be exhausted to the external environment, UV light has to be filtered out to avoid the production of ozone; elimination of the 185 nm line is a common feature of the lamps and is achieved in their manufacture by using appropriate glass materials.

The air flow characteristics through the duct are characterized by the dimensionless Reynolds number, Re, which is the ratio of inertial forces to viscous forces within a fluid [46]. It is defined by

(Eq. 18) Re=ρ u h/μ

where ρ is the density of the fluid, μ is its viscosity, u is the linear flow velocity, and h we take as the height of the flow duct; for u we divide the volumetric flow velocity by the cross-sectional area, A. This gives a value of Re = 5800. Typically, when Re <2300, the flow is smooth, laminar and predictable, and when Re >4000, the flow is turbulent. The turbulent flow for the simple design we have described will facilitate viral inactivation by UVGI since it will produce collisions of photons with viral lipids in the random way postulated above.

Adjusting the design parameters together with the light intensity along the duct, the level of viral inactivation and the time required for it can be varied to meet a required performance. That would be particularly useful if a greater level of inactivation than 3-log10 is needed and adjustments to compensate for the falloff in the deactivation rate have to be made.

In addition to the process chemistry and parameters that we have discussed, we should note, as mentioned earlier, that environmental factors, particularly heat and moisture, can have an effect on viral inactivation. At low to moderate temperatures that viruses will encounter in natural and artificial environments, heat primarily acts to break the O-O bonds of the hydroperoxides and this will accelerate the overall chain propagation and lipid oxidation [47].

Moisture and water add more complexity to lipid oxidation with both pro-oxidant and antioxidant effects. At very low relative humidities when aerosol water evaporates, thin moisture layers can bind to the macromolecular surface, which retards oxygen diffusion, and water can hydrogen bond to hydroperoxide. Both of those processes lead to decreasing oxidation. On the other hand, aerosol hydration of virus lipids increases molecular mobility, oxygen diffusion and interaction with the lipids [48].

The mechanism for the behavior is clearly intricate. However, being aware of the effects and of the chemistry involved can provide a basis for understanding the apparent conflicting results that have been obtained from geographical and healthcare settings [49].

Whatever the process parameters, once the required level of inactivation has been achieved, the treated air can be returned to the ward environment or exhausted to the external atmosphere.

## Conclusion

We have carried out an analysis of the inactivation of enveloped viruses in general and of SARS-CoV-19 in particular in terms of a suggested decomposition chemistry for the lipid bilayer through a free radical chain reaction. The analysis and the chemical considerations together with the application of the kinetics to other enveloped viruses indicate that the inactivations of such viruses are comparable.

Promoting virus inactivation with UV light has been briefly reviewed and discussed in terms of an extension of the chain reaction mechanism. Considerations of the chemistry also highlight why UV light can be a powerful initiator for virus inactivation, which together with physicochemical analyses give insights into how a 3-log10 level of deactivation in 1 min, or a greater level of inactivation, could be achieved.

A practical method for doing that has been outlined that could be used to supplement other engineering control methods and which would allow purified air to be returned to healthcare environments without there being any UV radiation hazards for personnel during the process. The procedure while having relevance to the present virus situation in a clinical setting, could have applications for mitigating viral transfer in commercial environments such as restaurants, retail outlets, entertainment venues and anywhere potential asymptomatic disease carriers gather in restricted spaces.

Finally, we should emphasize that a knowledge of viral lipid chemistry can be of value in bringing understanding and insight into the mechanisms of inactivation for COVID-19 aerosols.

## Future perspective

Although the current COVID-19 pandemic has led to hundreds of papers and articles about the causative agent and there have been rapid advances on various aspects of the disease, there is still much that is poorly understood. This will obviously require more clinically based research, but investigations of viral diseases should not be purely medically based. Viruses have complex molecular structures and their transfer to and interaction with living species inevitably involve chemical reactions and physicochemical processes. To better understand the epidemiology of viruses and to achieve new insights it will be necessary to have a wide perspective and to draw on a broad range of other scientific disciplines. And for that, future virology will need to have an open mind and for there to be a spirit of enterprise by the medical community.

**Executive summary**

**Virus inactivation & kinetics**

- Although there have been rapid advances on various aspects of the COVID-19, there is still much that is poorly understood. In this paper we use different perspectives to try and understand the chemistry of coronavirus aerosols and of viral inactivation.
- In some viruses the capsid of the virion with its enclosed nucleic acid is surrounded by a lipoprotein envelope, as in coronaviruses, and while such viruses can persist outside host environments typically for days, nonenveloped viruses can survive for weeks. It would not be unreasonable, therefore, to suggest that that could be related to the relative ease with which the lipid structure is disrupted.
- The most common mechanism of the degradation of lipids is environmental oxidation. The process by which that occurs is, in general, a free radical chain reaction leading to a series of complex chemical changes. Here we illustrate the process in a greatly simplified scheme of reactions in order to try and discover what could be involved in viral deactivation.
- It is shown that the kinetics of virus inactivation is consistent with a pseudo-first-order reaction and that it is a common feature of a range of viral envelopes.

**Promoting virus inactivation & use of UV light**

- The use of UV light to promote virus inactivation is reviewed and its effect on the oxidative chain reaction is discussed and illustrated with published data. However, while UV light can be a powerful promoter of virus inactivation, at least initially, the experimental temporal plots have decreasing slopes showing a slowing down of the inactivation processes. Various reasons for that are commented on, and it is shown that a model based on collisions between molecular species and photons can explain the effect.
- The use of UV light for viral inactivation in a practical way is then presented. Considerations of experimental parameters lead to a simple design that would allow contaminated air from a clinical setting to be extracted and treated in order to achieve a 3-log10 level of deactivation in 1 min so that purified air could be returned to healthcare environments.
